# Arterial Thrombosis in Patients with Human Immunodeficiency Virus: Two-Case Reports and Review of the Literature

**DOI:** 10.1155/2011/847241

**Published:** 2011-09-12

**Authors:** C. Konin, J. B. Anzouan-Kacou, A. Essam N'loo

**Affiliations:** Abidjan Cardiology Institute, BP 487, Abibjan 22, Cote D'Ivoire

## Abstract

Thrombosis during HIV infection was commonly vein thrombosis. Arterial thrombosis is also more and more described. We report two cases detected in the Abidjan Cardiology Institute. *Case Reports*. Case 1: an HIV infected female presented with sudden loss of consciousness and right hemiplegia. She had been taking HAART regimen for five years. Neck vessels ultrasonography revealed thrombosis on left ICA. Anticoagulant treatment leads to reduction of symptoms and left ICA partial recanalization. Case 2: male HIV infected taking HAART therapy was admitted for an acute pain of left lower limb; examination showed a decrease of heat, sensitivity, and mobility of this limb with popliteal and tibial pulses abolished. Arterial ultrasonography and CT angiography showed occlusion on the lower third of superficial femoral artery and homolateral popliteal artery suggesting a thrombosis of this artery. He underwent a femorotibial bypass surgery and anticoagulant treatment. The outcome was good with reappearance of local heat of the limb and tibial pulses. Probable etiology is early carotid atherosclerosis associated with protein S deficiency in the first case and antiphospholipid syndrome in the second case. *Conclusion*. Arterial thrombosis might occur in HIV infection. Several etiological factors could be involved in the pathogeny of these arterial thromboses.

## 1. Introduction

HIV infection is well characterized by its multivisceral aspects. Cardiovascular system is known to be involved in this disease [1]. Different forms of cardiovascular manifestations are described as thrombosis. Thrombosis during HIV infection is commonly vein thrombosis. Arterial thrombosis nowadays is also more and more described [2]. Pathogeny of this arterial thrombosis is still not well known [1]. Etiopathogeny involves many aspects sometimes associated. We report two cases of multiple arterial thrombosis with different complex etiopathogenic mechanisms involving two patients with HIV infection. These patients are being treated with antiretroviral (ARV) drugs.

## 2. Case  1

A woman of 42, was hospitalized from the 5th to the 21st of September 2010 at the Abidjan Cardiology Institute, for the left internal carotid artery (ICA) thrombosis. Symptoms started a couple of days before admission with sudden loss of consciousness and right hemiplegia. This patient was undergoing a followup from April 2003 for HIV type 1 and 2 infection. She was taking second-line ARV drugs, Abacavir, didanosin, lopinavir, and Ritonavir since August 2005. She developed metabolic syndrome just after having started taking ARV drugs. This syndrome was made of dyslipidemia, hypertension, and lipodystrophia and was treated with atorvastatin 10 mg per day and captopril 50 mg per day. She reported no personal or family history of thrombosis disease.

Physical examination revealed a pyramidal syndrome with a muscle power 0 out of 5 for upper and lower limbs. Cephalic tomodensitometry revealed a left cerebral infarction without any infectious nor bleeding lesion. Ultrasonography of the neck vessels showed a thrombosis on left ICA, B-dimensional showing isoechogenic contrast inside left ICA, and Doppler's mode showing a lack of blood flow in ICA, a reversed flow in the left ophthalmic artery, and a resistive flow on the left common carotid artery (CCA) with a resistance index 1. Ultrasonography also showed atherosclerosis of carotid bifurcations with small hyperechogenic plaques. ECG showed sinus tachycardia 120 beats per min, ST inversion in anterolateral territory. Cardiac ultrasound was normal. There was no intracardiac thrombus at transoesophageal echocardiography. Hemogram showed hemoglobin (Hb) 11 g/dl, platelet count 173000/mm^3^, white blood cells (WBC) 5870/mm^3^. Hemostasis was normal with a prothrombin ratio (PR) 100% and activated partial thromboplastin time (aPTT) 33 seconds for the patient as for the sample. Antiphospholipid antibodies rate was normal. Venereal disease research laboratory test (VDRL) was negative. The CD4 count was 645/mm^3^ and viral load undetectable (detection threshold = 100/mm^3^). However, total cholesterol was 2.17 g/l, HDLc 0.56 g/l, LDLc 1.51 g/l, and triglycerides 0.47 g/l. That patient was given intravenous heparin before a switch to acenocoumarol. The outcome was characterized by regression of hemiplegia with hemiparesis as sequels with a muscle power 3 out of 5. Ultrasonography showed partial recanalisation of left ICA. Motility, sensitivity, and coloration of the foot were normal. Vascular ultrasonography showed an intraluminal isoechogenic contrast with some areas of partial recanalisation as a result of poplitea arteries and left and right tibial-peroneal trunks thrombosis. There was a decreased Doppler's flow in tibial arteries.

## 3. Case  2

Man of 42 with history of a treated Buruli's ulcer since he was 12 years old presented with retractile scars on left feet and on right hand. He is HIV infected treated with ARV drugs AZT, 3TC, NVP as from February 2009. He had a bad compliance to the drug therapy. He was admitted as an emergency on 23rd October 2010 at the Abidjan Cardiology Institute. He was suffering from an acute pain of left lower limb. This pain, which suddenly occurred 3 days before admission, was permanent and was causing sleeplessness. Physical examination showed diminution of the heat of left lower limb with popliteal and tibial pulses abolished. Color of the limb was normal. 

There was a decrease of sensitivity and motility. Blood pressure was 130/80 mmhg and pulse 88 beat per minute. Arterial ultrasonography and CT angiography showed occlusion on the lower third of superficial femoral artery and homolateral popliteal artery suggesting a thrombosis of these arteries. As an emergency, he underwent a femoro-tibial bypass surgery. The medical treatment was made of intravenous heparin before a switch with clopidogrel per os 75 mg per day. The outcome was good with reappearance of local heat of the limb and tibial pulses. Biology showed Hb 12 g/dl, platelets count 212000/mm^3^, PR was 100%, aPTT 40 second for the patient, 33 sec for the sample. Etiological research revealed no protein C or S nor antithrombin deficiency. Research of factor V leiden gene mutation was negative.

Nevertheless, we found a high rate of IgG anticardiolipin autoantibodies 89 GPL/ml (normal value <10 GPL/ml) and a high rate of IgG antinative DNA autoantibodies 47 UI/ml (normal value <20 UI/ml). VDRL was negative. Fibrinemia was 3.9 g/l (2–4.5 g/l), ESR 27 mm at 1st hour. The CD4 count was 155/mm^3^ and viral load 242,000 copies/mm^3^.

The patient got out of hospital on the 28th of October 2010. He was readmitted on 4th of November 2010 presenting left leg swelling. Doppler's ultrasonography showed a deep vein thrombosis involving left suropopliteal vessels with thrombus head in the third lower of superficial femoral vein. The patient received an anticoagulant treatment made of low-molecular-weight heparin switched with acenocoumarol. Outcome was good.

## 4. Discussion

The prevalence of thrombosis in HIV infection is increasing, due to the use of HAART regimen including protease inhibitors (PI). These thromboses involve arterial, venous circulation and microcirculation. 

### 4.1. Frequency of Arterial Thrombosis in HIV Infection

There are no arterial thrombosis large-cohort studies in the literature. Some cases with small cohort with low frequencies have just been reported [3].

### 4.2. Arterial Thrombosis Localization in HIV Infection

HIV infection is a risk factor of arterial and venous thrombosis. Arterial lesions are reported to involve commonly small arteries. However, thrombosis could involve large arterial trunks in HIV infection [4].

### 4.3. Thrombosis Physiopathology in HIV Infection

Thrombosis physiopathological mechanism, particularly arterial thrombosis in HIV infection, is not yet well known. Several mixed etiological factors are reported. These factors might lead to hemostasis disorders as hypercoagulability. Among these factors, there are cytomegalovirus opportunistic infections, thrombogenic contribution of HIV infection due to the von Willebrand factor increase, C and S proteins decrease, deficiency of heparin cofactor II [5]. ARV drugs such as PI might lead to thrombosis. The chronic infection of HIV-infected individuals promotes chronic arterial inflammation and injury, which, in turn, promotes dysfunction of the endothelium, atherosclerosis, and thrombosis. Endothelial injury and dysfunction have been proposed as plausible links between HIV-infection and atherosclerosis [6, 7]. The development of atherosclerosis may be the consequence of infection-triggered endothelial damage, and atherosclerotic cardiovascular events are commonly manifested via thrombotic events. Antiphospholipid antibodies found in HIV-infected people might lead to thrombosis even though thrombogenic contribution of HIV infection is not well admitted [8]. The common thread through these 2 cases is arterial thrombosis in HIV infected people taking HAART for several months: 7 years and 5 months for the 1st case and 20 months for the 2nd. However, physiopathology of these arterial thromboses seems to be different among these cases. 

Our first case was a 42-year-old HIV-infected woman taking ARV drug for more than 7 years. She developed a metabolic treatment complication including hypercholesterolemia, lipodystrophia and hypertension. This metabolic complication led to an early carotid atherosclerosis in this African woman without any personal cardiovascular risk factors nor family history. Metabolic dysfunction with early atherosclerosis has been reported in HIV-infected patients taking ARV drugs particularly protease inhibitors [9, 10]. Although not being unanimous [11], these disturbances of lipid metabolism are responsible for a significant increase in the risk of coronary atherosclerosis by multiplying by 4 the risk of myocardial infarction and by 5 the risk of cardiovascular mortality [12]. A possible plaque rupture with activation of coagulation cascade might be at the beginning of carotid thrombus formation. This process seems to have been reinforced by deficiency of protein S and protease inhibitors thrombogen effects. Protein S deficiency was moderate but was able to lead to thrombogen effects. There was no personal or family history of thrombophilia. This deficiency might be an acquired protein S deficiency due to HIV infection or ARV drugs. The causes of carotid thrombosis might be combined effects of carotid atherosclerosis, protein S deficiency, and protease inhibitors thrombogen effects.

Three main mechanisms could explain thrombosis physiopathology of the 2nd patient: HIV effect itself on vascular endothelium, ARV drugs thrombogenic contribution, and antiphospholipid syndrome. Arterial and venous thrombosis when having lupus-circulating anticoagulant and increase of anticardiolipin antibodies lead to the diagnosis of antiphospholipid syndrome [13]. Antiphospholipid antibodies such as anticardiolipin are frequently found in HIV patients [8, 14, 15]. However, it appears that there is no risk to develop arterial or venous thrombosis, although some thrombotic events have been reported. Most of these events are “anti-*β*2-glycoprotéineI independent” and without any thrombogenic capacity [8]. These antibodies could mean an intense and/or chronic antigenic stimulation of immune system [8]. However, the combined effect of the presence of antiphospholipid syndrome and viral replication associated with poor adherence in patients taking HAART treatment for several months could increase risk of thrombosis.

## 5. Conclusion

These 2 cases show that arterial thrombosis might occur in patients with HIV infection. Several etiological factors could be involved in the pathogeny of these arterial thromboses. More studies including more cases, particularly in Sub-Saharan Africa where HIV infection is endemic, are to be done in order to reveal all the etiopathogenic hypothesis.

## 6. Summary

Authors report two cases of great trunks' arterial thrombosis in patients who are HIV infected and have been taking ARV drugs for several months. Probable etiology is early carotid atherosclerosis associated whith protein S deficiency in the first case and antiphospholipid syndrome in the second case (Table 1).

## Figures and Tables

**Table 1:** Summary of the two cases.

Characteristics	Case 1	Case 2
Age (years)	42	42
Sex	F	M
Personal and family histories	None	Buruli's ulcer
ARV start	2003	2009
Type ARV	Abc + ddl + lopinavir + ritonavir	AZT, 3TC, NVP
ARV's other complications	Dyslipidemia, hypertension, lipodystrophia	None
Hospitalization period	5th–21st September 2010	23rd–28th October 2010
Examination	Right pyramidal syndrome	Subacute limb ischaemia
Others signs	None	None
Thrombus localization	left ICA	FSA, PA, FSV, left PV
Others Doppler lesions	Carotid atherosclerosis	None
Biological syndrome associated	Protein S deficiency	Antiphospholipid syndrome
CD4 count/mm^3^	645	155
viral load(copies/mm^3^)	Undetectable	242,000
Treatment	UFH, AVK	UFH, aspirin, AVK
Evolution	Partial recanalization, motor sequels	Good

SFA: superficial femoral artery, SFV: superficial femoral vein, PA: popliteal artery, PV: popliteal vein, ICA: internal carotid artery, LV: left ventricular, UFH: unfractionated heparin, AVK: antivitamin K, AZT: zidovudin, Abc: abacavir, ddl: didanosin, 3TC: lamivudin, and NVP: névirapin.
